# Cytotoxic Activity against A549 Human Lung Cancer Cells and ADMET Analysis of New Pyrazole Derivatives

**DOI:** 10.3390/ijms22136692

**Published:** 2021-06-22

**Authors:** Agnieszka Czylkowska, Małgorzata Szczesio, Anita Raducka, Bartłomiej Rogalewicz, Paweł Kręcisz, Kamila Czarnecka, Paweł Szymański, Monika Pitucha, Tomasz Pawlak

**Affiliations:** 1 Institute of General and Ecological Chemistry, Faculty of Chemistry, Lodz University of Technology, 90-924 Lodz, Poland; malgorzata.szczesio@p.lodz.pl (M.S.); anita.raducka@edu.p.lodz.pl (A.R.); 211150@edu.p.lodz.pl (B.R.); 2 Department of Pharmaceutical Chemistry, Drug Analyses and Radiopharmacy, Faculty of Pharmacy, Medical University of Lodz, 90-151 Lodz, Poland; pawel.krecisz@umed.lodz.pl (P.K.); kamila.czarnecka@umed.lodz.pl (K.C.); pawel.szymanski@umed.lodz.pl (P.S.); 3 Department of Radiobiology and Radiation Protection, Military Institute of Hygiene and Epidemiology, 01-163 Warsaw, Poland; 4 Independent Radiopharmacy Unit, Faculty of Pharmacy, Medical University of Lublin, 20-093 Lublin, Poland; monika.pitucha@umlub.pl; 5 Centre of Molecular and Macromolecular Studies, Polish Academy of Sciences, 90-363 Lodz, Poland; tpawlak@cbmm.lodz.pl

**Keywords:** cytotoxic activity, pyrazole derivatives, MTT assay, ADMET analysis, single-crystal diffraction, FTIR spectroscopy, NMR spectroscopy thermogravimetric analysis

## Abstract

Two new pyrazole derivatives, namely compound **1** and compound **2,** have been synthesized, and their biological activity has been evaluated. Monocrystals of the obtained compounds were thoroughly investigated using single-crystal X-ray diffraction analysis, FTIR spectroscopy, and NMR spectroscopy. The results gathered from all three techniques are in good agreement, provide complete information about the structures of **1** and **2**, and confirm their high purity. Thermal properties were studied using thermogravimetric analysis; both **1** and **2** are stable at room temperature. In order to better characterize **1** and **2**, some physicochemical and biological properties have been evaluated using ADMET analysis. The cytotoxic activity of both compounds was determined using the MTT assay on the A549 cell line in comparison with etoposide. It was determined that compound **2** was effective in the inhibition of human lung adenocarcinoma cell growth and may be a promising compound for the treatment of lung cancer.

## 1. Introduction

There are many different types of compounds, both natural and synthetic, with potential medical properties. Substances, such as imidazoles, oxadiazoles, pyrroles, and many of their derivatives, are well studied and described in the literature [1,2,3,4,5]. The main goal of medicinal chemistry is to synthesize compounds with promising activity and therapeutic agents that exhibit lower toxicity. The search for new, pharmacologically active chemical compounds is related to the modification of existing molecules. A group of compounds that is potentially interesting, due to its structure and biological activity, is those substances containing a pyrazole ring. Pyrazoles exist in many compounds that are used as pharmaceuticals and other active compounds [6,7,8]. Diseases caused by microbial infection are a serious menace to the health of human beings and often are connected with some other diseases whenever the body system becomes debilitated. The growing incidence of microorganisms that resist antimicrobials is a constant concern for the scientific community. Pyrazoles have always been considered as a scaffold-of-choice in designing novel therapeutic agents because of their anti-inflammatory, anti-tumor, antihyperglycemic, antipyretic, analgesic, antibacterial, and antifungal properties [9,10,11,12,13,14,15,16]. In this work, we present a synthesis, structural characterization, and investigation of the biological, spectroscopic, and thermal properties of two new pyrazole derivatives. The compounds were also tested for cellular survival through MTT assays on the A549 cell line, which investigated their biological properties.

## 2. Results and Discussion

### 2.1. Synthesis

**Scheme 1:**
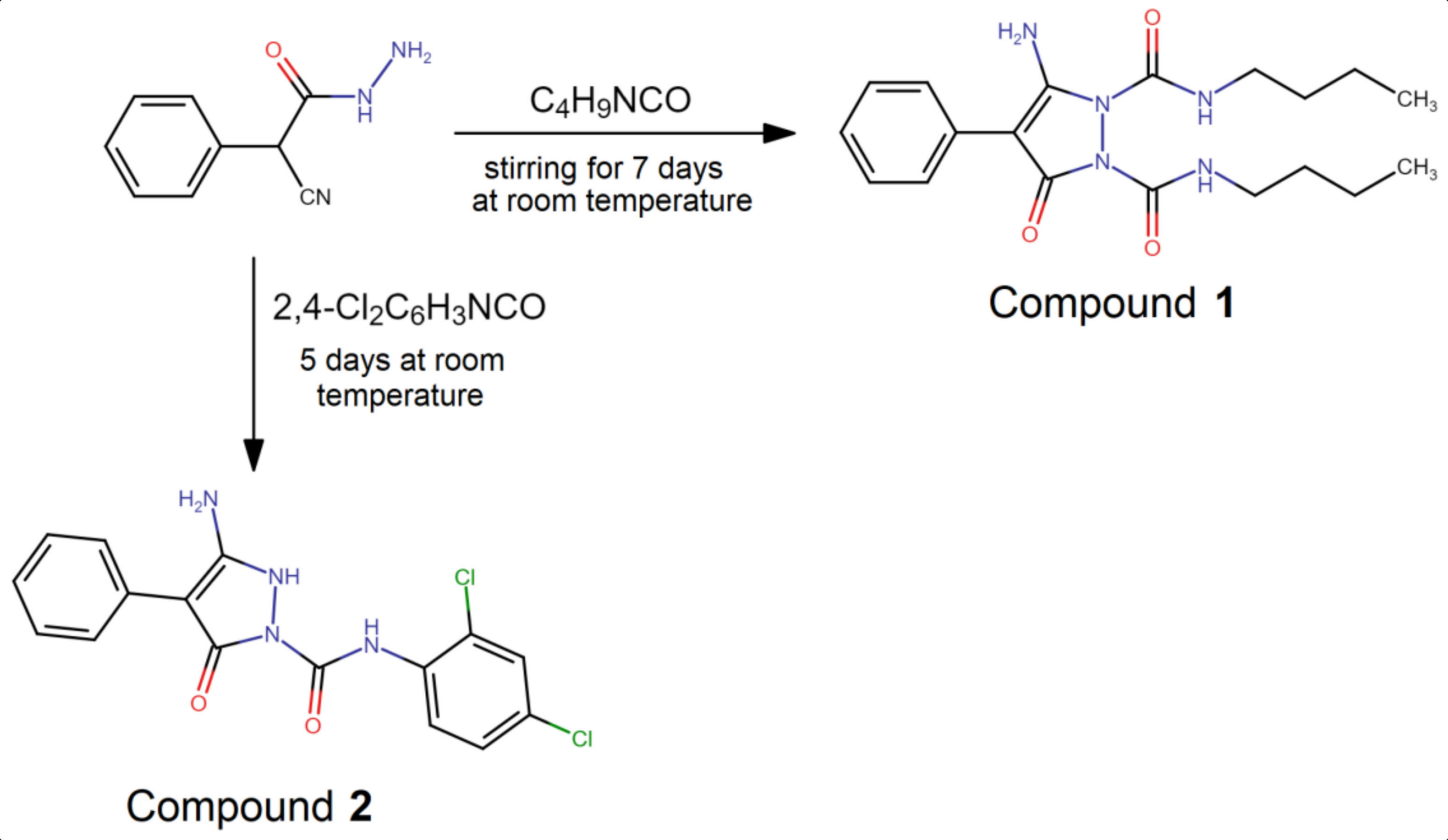
Synthesis route of the obtained pyrazole derivatives.

The title compounds **1** and **2** have been synthesized in the reaction of 1-cyanophenyl acetic acid hydrazide with isocyanates (butyl and 2,4-dichlorophenyl), according to Scheme 1.

Compound **1**: 3-amino-*N*^1^,*N*^2^-dibutyl-5-oxo-4-phenyl-pyrazole-1,2-dicarboxamide.

To synthesize compound **1**, 1.75 g of 1-cyanophenylacetic acid hydrazide (0.01 mol) was dissolved while hot and in 15 mL of anhydrous acetonitrile. Then, 1.98 g (0.02 mol) of butyl isocyanate was added to the warm mixture. Stirring was allowed for 7 days at room temperature. The precipitate was then filtered off and dried. The reaction yield was 67%, and the melting point was 131.1 °C.

Compound **2**: 3-amino-*N*-(2,4-dichlorophenyl)-5-oxo-4-phenyl-2H-pyrrole-1-carboxamide.

To synthesize compound **2**, 1.75 g of 1-cyanophenylacetic acid hydrazide (0.01 mol) was dissolved in 15 mL of anhydrous acetonitrile. Then, 1.88 g (0.01 mol) of 2,4-dichlorophenyl isocyanate was added. The mixture was kept at room temperature for 5 days. The precipitate was then filtered off and dried. The reaction yield was 81%, and the melting point was 188.9 °C.

### 2.2. Crystal Structure Analysis

**Figure 1:**
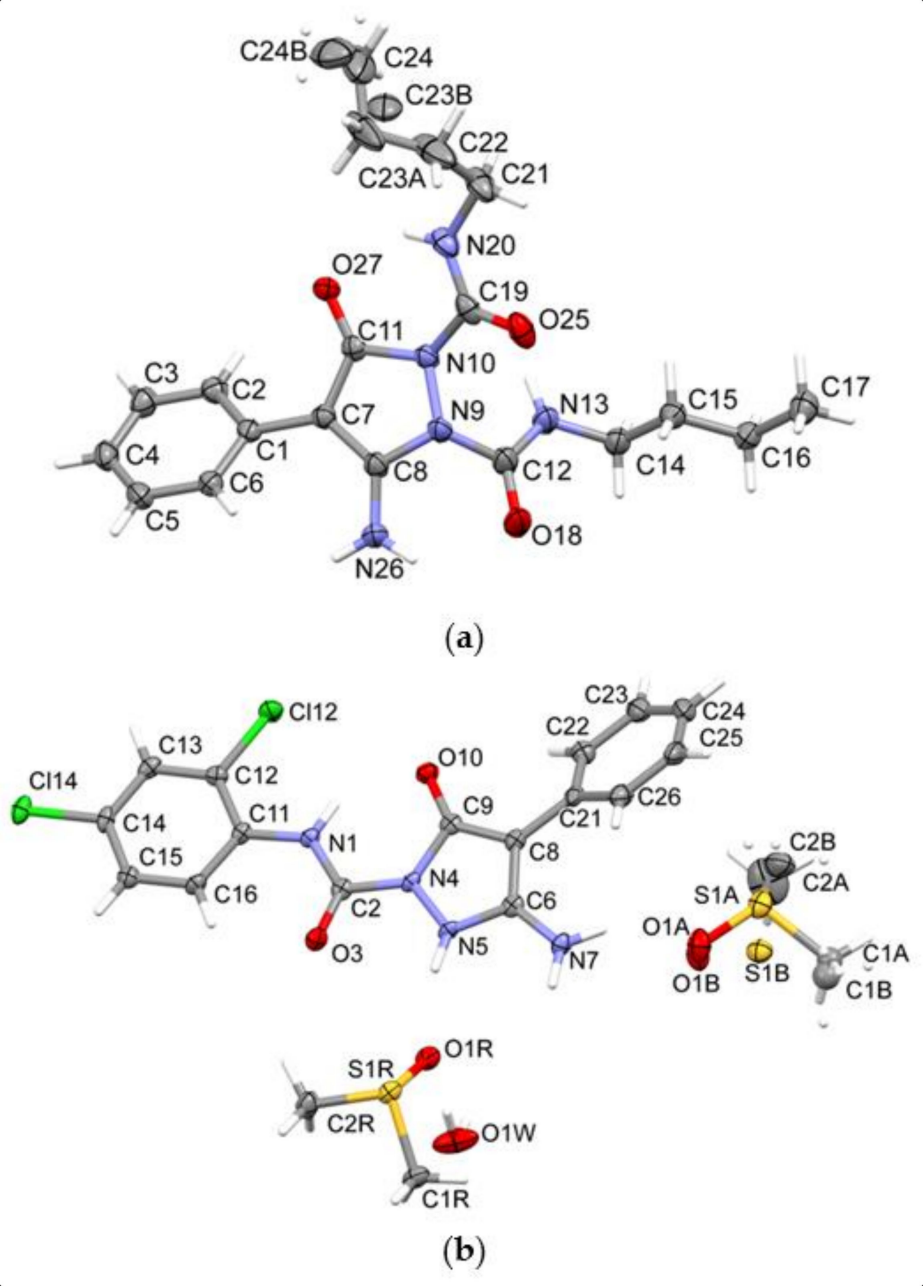
The molecular structure and atom-numbering schemes for: compound **1**—figure (**a**), and compound **2**— figure (**b**), with displacement ellipsoids drawn at the 50% probability level.

Both structures crystallized in the P2_1_/c space group in a monoclinic crystal system. The crystal data and structure refinement details are summarized in Table 1. Compound **1,** crystallized from CH_3_OH, has one molecule in the asymmetric *unit* (Figure 1a). One of the butyl fragments shows a disorder. The single crystal of compound **2** crystallized from DMSO. In this case, the molecules of solvents are built into the structure; this involves two DMSO molecules, including one disordered and one water molecule (Figure 1b). Tautomerism was described in an earlier publication from 2014 containing an analogous compound [17]. Compound **2** adopts a keto tautomeric form in a crystalline state. There are typical carbonyl bonds in the structure (C2-O3 = 1.221(4) Å, C9-O10 = 1.242(4) Å). Compound **1** also contains only typical carbonyl groups.

**Figure 2:**
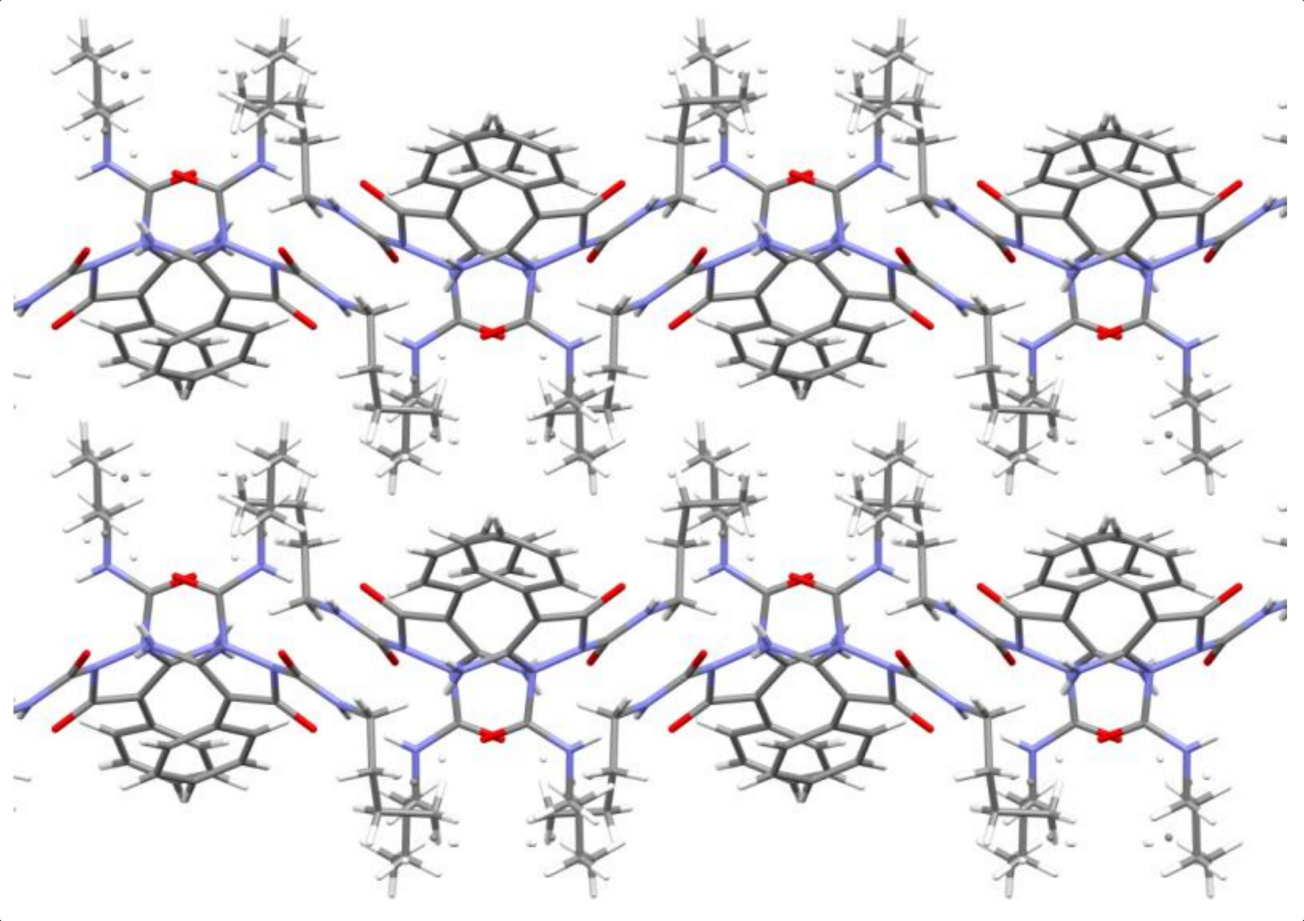
The crystal packing of **1**, viewed along the c axis.

The conformation of the molecules is stabilized by intra-molecular hydrogen bonds (S(6)), according to the theory of Bernstein [18]. These are N-H…O bonds (N26—H26B⋯O18 and N20—H20⋯O27) (Table 2). The packing of the molecules of the structure **1** is layered (Figure 2). These layers are stabilized by chain hydrogen bonds N26—H26A ⋯ O25 (C (7)—graph-set) and additionally form dimers N13—H13⋯O27 (R_2_^2^(14)).

**Figure 3:**
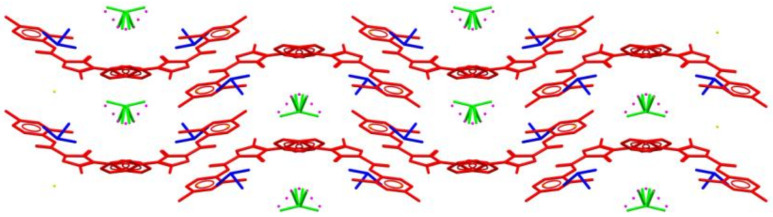
The crystal packing of **2**, viewed along the c axis.

The molecules in structure **2** create gaps into which the disordered solvent molecules are fit (Figure 3). The DMSO molecules form hydrogen bonds of the N-H… O(DMSO) type. A water molecule stabilizes the structure with three hydrogen bonds with two **2** molecules and one DMSO molecule (Table 3).

### 2.3. NMR Studies

It is well known that monocrystals selected for single-crystal X-ray analysis do not always represent the bulk material. Therefore, we additionally performed a validation analysis of the studied materials using the solution NMR technique (Figures S1 and S2 are in the Supplementary Materials). For both compounds **1** and **2,** we recorded series of NMR spectra as ^1^H, ^13^C, ^1^H-^1^H COSY, ^1^H-^13^C HSQC, and ^1^H-^13^C HMBC, and made full assignments of ^1^H as well as ^13^C signals. The following NMR parameters were noted for studied compounds: compound **1**, ^1^H (DMSO-d_6_) δ [ppm]: 0.87 (3 × H17, 3 × H24), 1.30 (2 × H16, 2 × H23), 1.44 (2 × H15, 2 × H22), 3.10–3.20 (2 × H14, 2 × H21), 7.20 (H4), 7.28 (H26), 7.37 (H3, H5), 7.49 (H2, H6), 8.10–8.26 (H13, H20) and ^13^C (DMSO-d_6_) δ [ppm]: 13.5–13.6 (C17, C24), 19.2–19.3 (C16–C23), 30.7–31.2 (C15, C22), 39.2–40.1 (C14, C21), 86.0 (C7), 125.6 (C4), 127.3 (C3, C5), 128.2 (C2, C6), 130.2 (C1), 150.5–152.7 (C12, C19), 156.0 (C8), 165.6 (C11); compound **2**, ^1^H (DMSO-d_6_) δ [ppm]: 6.90 (2 × H7), 7.16 (H24), 7.34–7.37 (H23, H25), 7.46 (H15), 7.56 (H22, H26), 7.70 (H13), 8.31 (H16), 10.81 (H5), 11.73 (H1) and ^13^C (DMSO-d_6_) δ [ppm]: 85.9 (C8), 121.6 (C16), 122.9 (C21), 124.9 (C24), 126.6 (C22, C26), 127.1 (C12), 147.1 (C2), 128.0 (C15), 128.2 (C23, C25), 128.8 (C13), 131.4 (C14), 134.3 (C11), 156.3 (C6), 163.2 (C9). Apart from the listed resonances, the residual DMSO, as well as the H_2_O and HOD signals, were clearly visible on the spectra. The results obtained herein confirmed that both the **1** and **2** samples are homogenous, pure, and stable in DMSO solution. The single-crystal structures presented in the previous section are fully consistent with the NMR results.

### 2.4. FTIR Spectra

**Figure 4:**
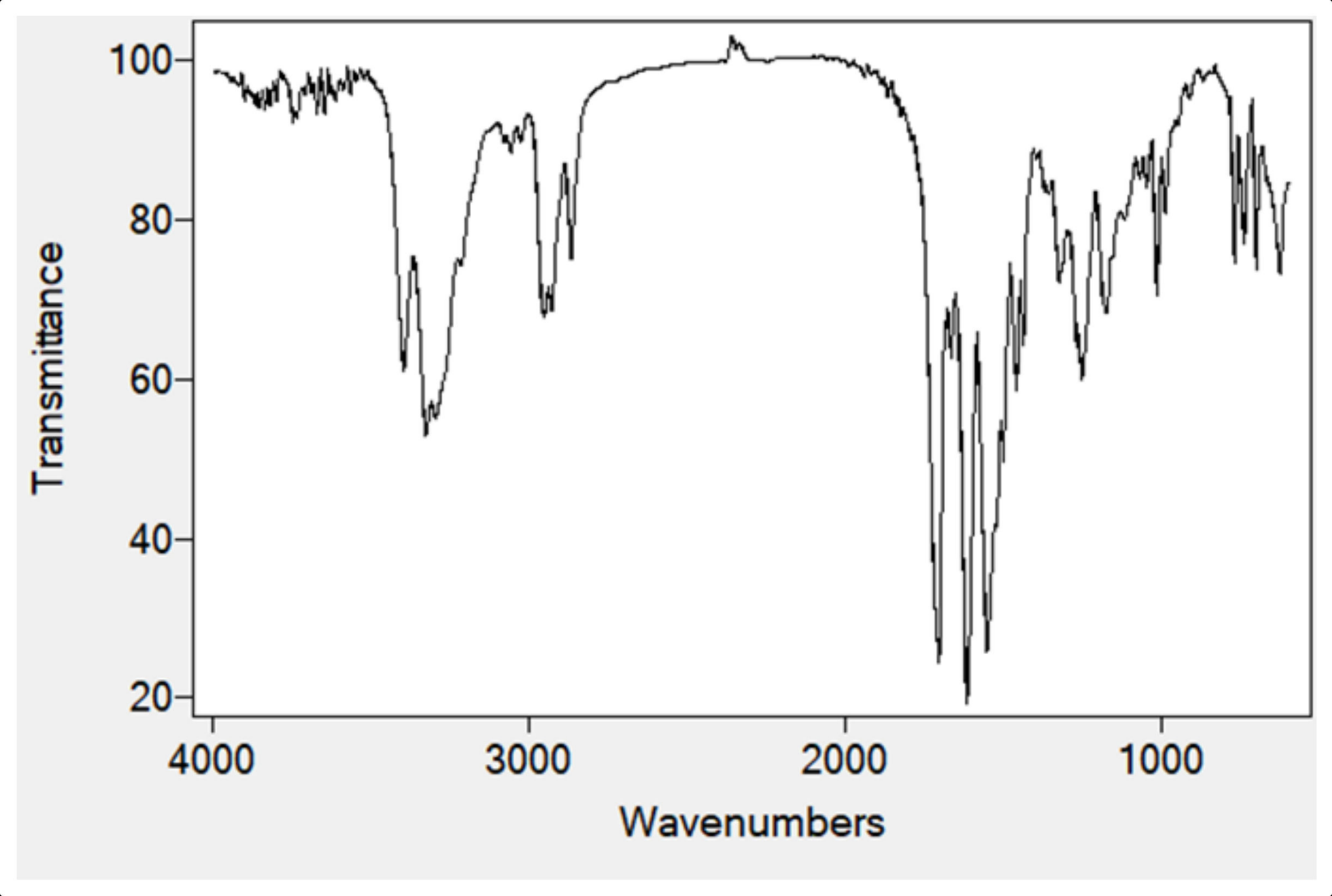
FTIR spectrum of compound **1**.

**Figure 5:**
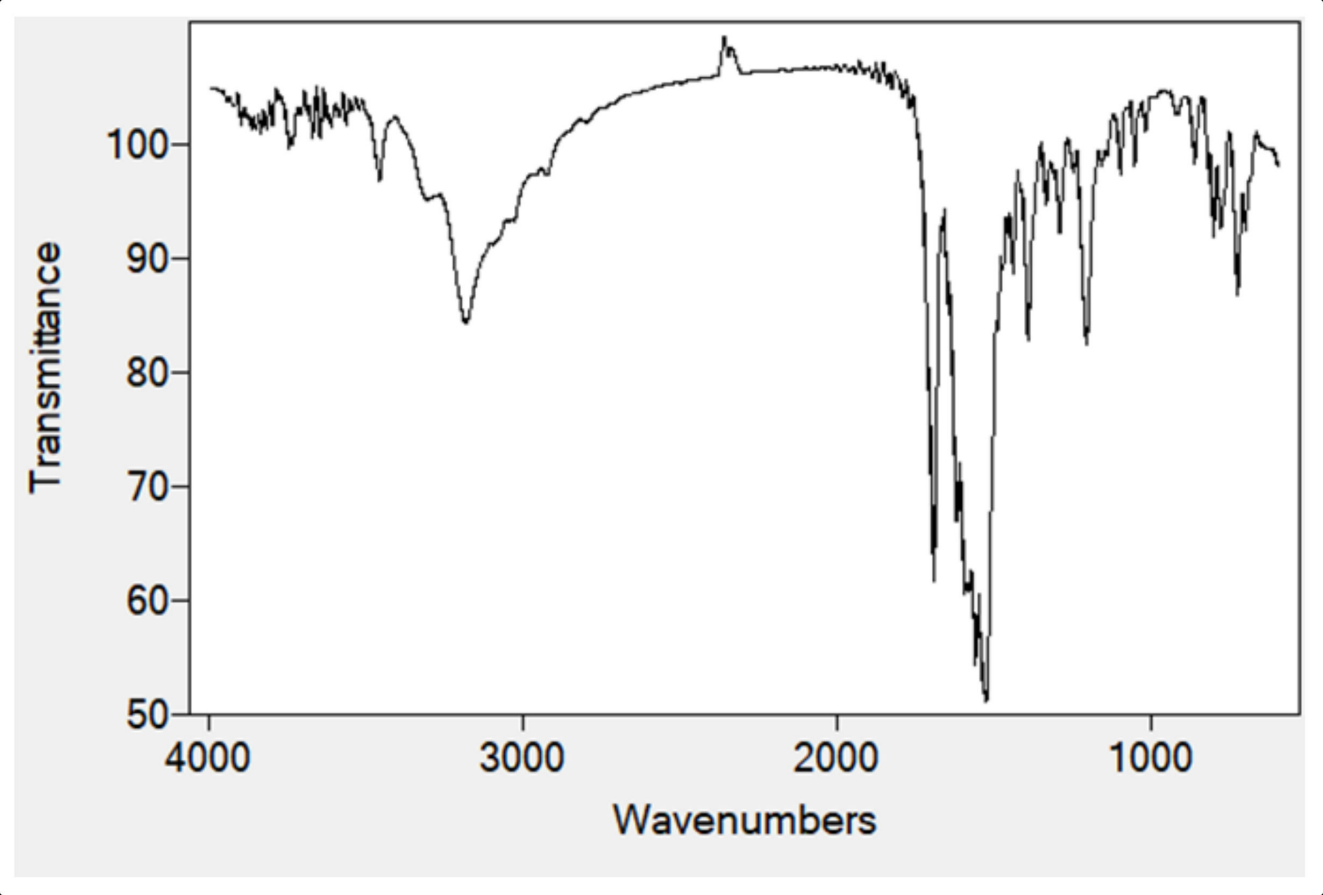
FTIR spectrum of compound **2**.

The FTIR spectra of both compounds confirm their molecular structures. Both spectra (Figure 4 and Figure 5) show several bands of various intensities and shapes that can be ascribed to *ν*(NH) in the range of 3500–3100 cm^−1^—with the sharpest peaks at 3398, 3328, and 3297 cm^−1^ for **1** and 3459, 3308, and 3184 cm^−1^ for **2**, respectively. Due to the presence of a butyl group in the **1** molecule, we can observe characteristic bands in the range of 3090–2850 cm^−1^ that correspond to alkane *ν*(CH) modes, with peaks at 3081, 3060, 3028, 2954, 2931, and 2869 cm^−1^. In both spectra, we can observe sharp bands in the range of 1730–1680 cm^−1^ that result from *ν*(C=O) modes—in the **1** spectrum at 1707 cm^−1^ and in the **2** spectrum at 1710 and 1694 cm^−1^. In both spectra, there are several bands present in the 1630–1500 cm^−1^ region that can be ascribed to *ν*(CN), *ν*(C=C), and *δ*(NH) modes. Sharp bands in the **1** spectrum at 1461 and 1440 cm^−1^ most likely correspond to the *δ*(CH) methyl group modes. When moving to the lower wavenumbers, we can observe bands for both compounds in ranges of 1280–1110 cm^−1^ and 790–690 cm^−1^ that correspond to *β*(CH) and *γ*(CH) modes, respectively. Several sharp bands that result from *ν*(NN) vibrations can also be observed in the **1** spectrum (1050 and 1016 cm^−1^) and in the **2** spectrum (1055, 1019 cm^−1^). The bands that are present in the 870–790 cm^−1^ range in the **2** spectrum, but are absent in the **1** spectrum, can most likely be assigned to *ν*(CCl).

### 2.5. Thermogravimetric Studies in Air

**Figure 6:**
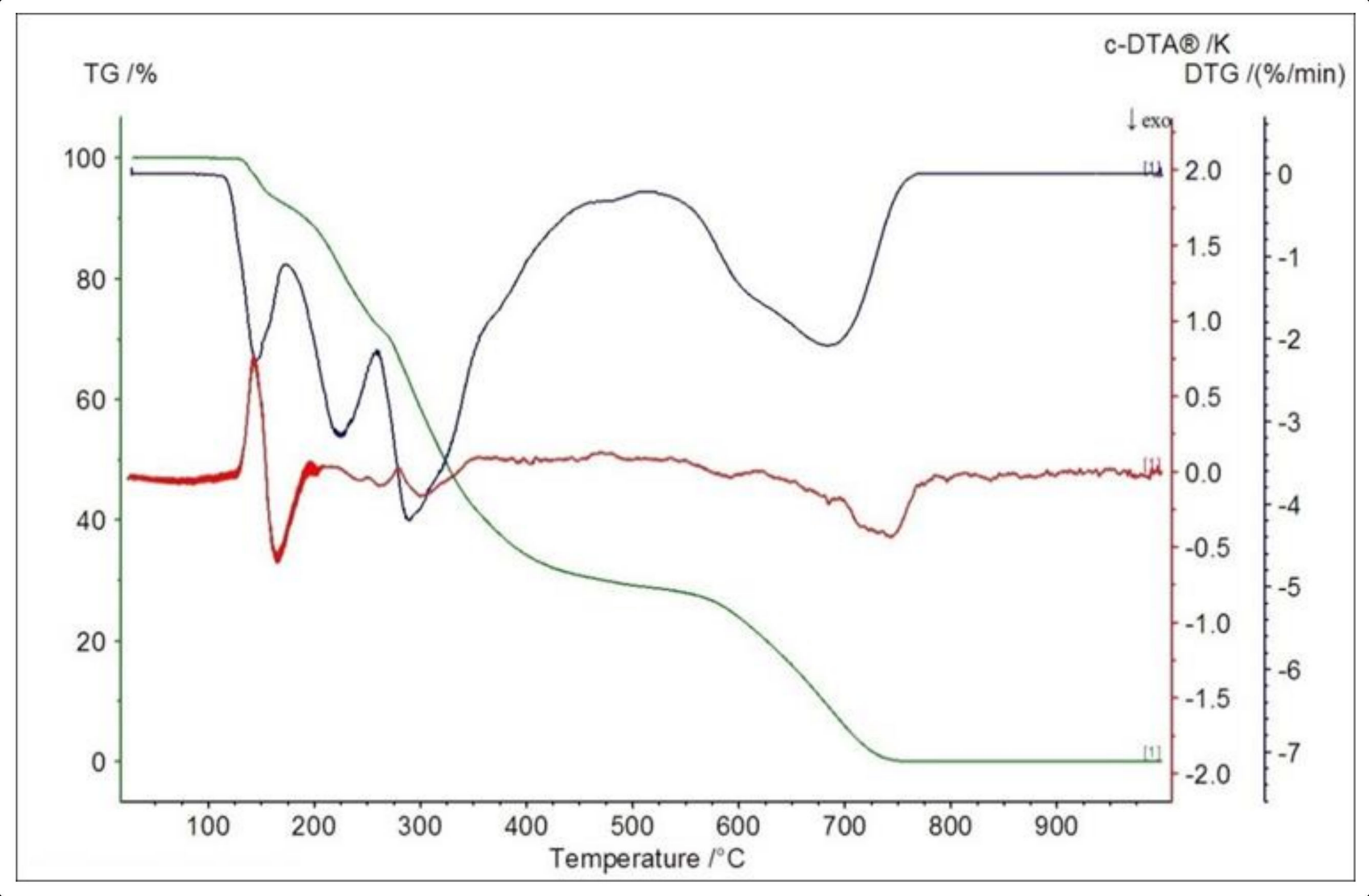
Thermal decomposition of compound **1**.

**Figure 7:**
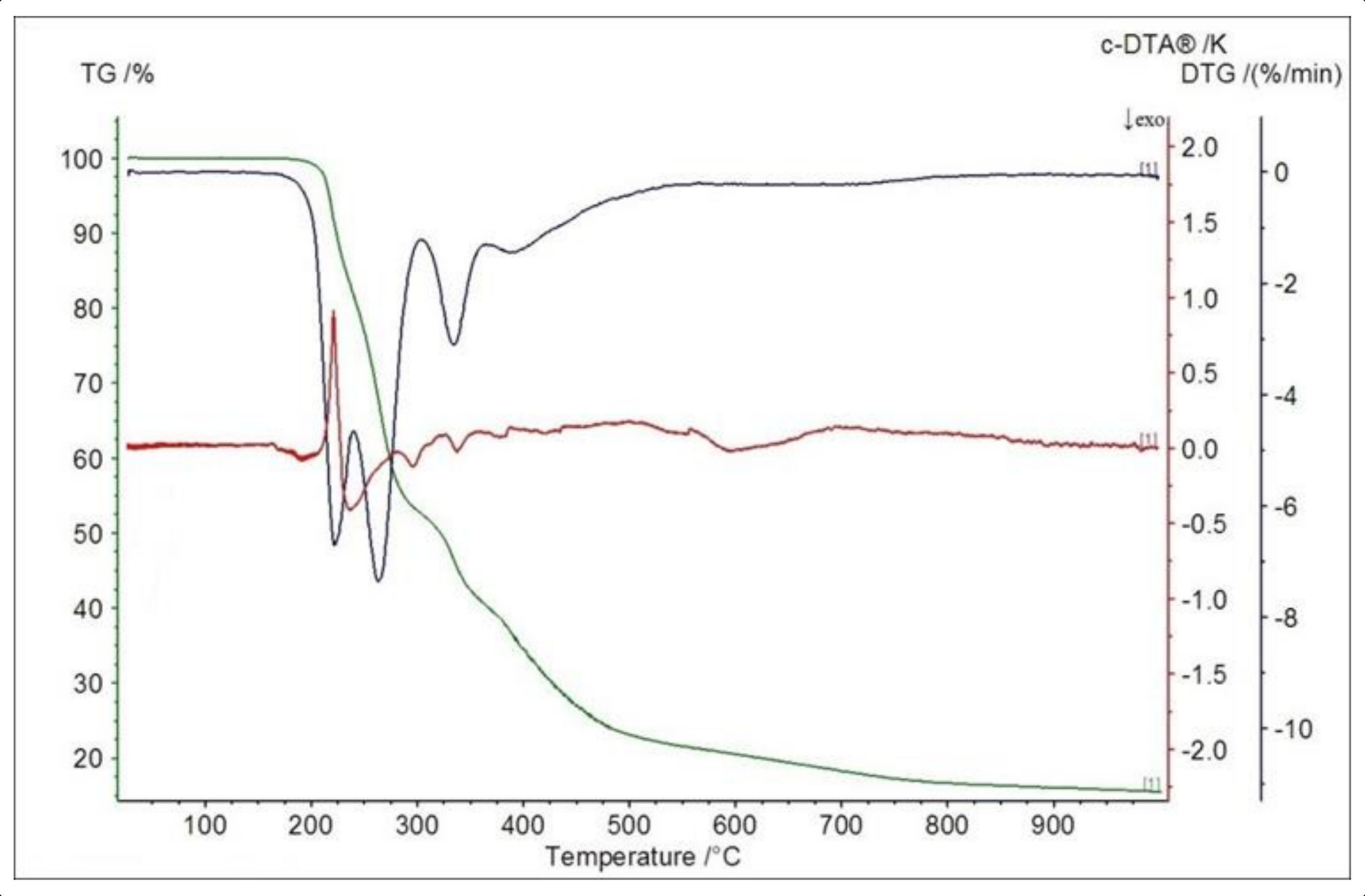
Thermal decomposition of compound **2**.

The thermal decomposition of **1** is shown in Figure 6. This compound is thermally stable up to 125 °C. In the first stage of thermolysis, one of the aliphatic chains is destroyed. In the temperature range of 125–175, the exothermic effect on the DTA curve is observed (175 °C). When the temperature rises, further destruction of **1** takes place. On the TG curve, there are three mass losses of 8.0% (calc. 7.78%), 19.0% (calc. 19.32%), and 40.0% (calc. 39.68%) within the temperature ranges of 125–175 °C, 175–250 °C, and 250–525 °C, respectively. The final step of decomposition is the burning of organic residues with corresponding exothermic effects on the DTA curve at 680 °C. In Figure 7, the TG, DTG, and DTA curves of **2** are shown. Compound **2** starts to decompose at 175 °C. The first step of pyrolysis is the destruction of the benzene ring. This process is accompanied by an exothermic peak on the DTA curve at 225 °C. The thermolysis of **2** is also a multi-stage and overlapping process. In the temperature range of 175–240 °C, the experimental mass loss is 21.0% and it is calculated at 21.23%. The next step is connected with the 52.0% (calc. 52.06%) loss of mass and occurs between 240 and 300 °C; on the DTA there are peaks at 270 °C, and when the temperature rises above 900 °C, the process stops.

### 2.6. Biological Assays

In A549 cells, the 50% effective concentration (EC_50_) for compounds **1** and **2** was found to be 613.22 and 220.20 µM, respectively. The values of the effective concentration after the treatment of the compounds are given in Table 4. It was observed that all synthesized molecules were very active; compound **1** showed much less toxicity than **2**. These results revealed that **2** showed the highest cytotoxicity and the most significant decrease in cell viability relative to the A549 lung cancer cell line. This is very interesting, as both compounds are active in between the activities of the etoposide.

### 2.7. ADMET Analysis

**Figure 8:**
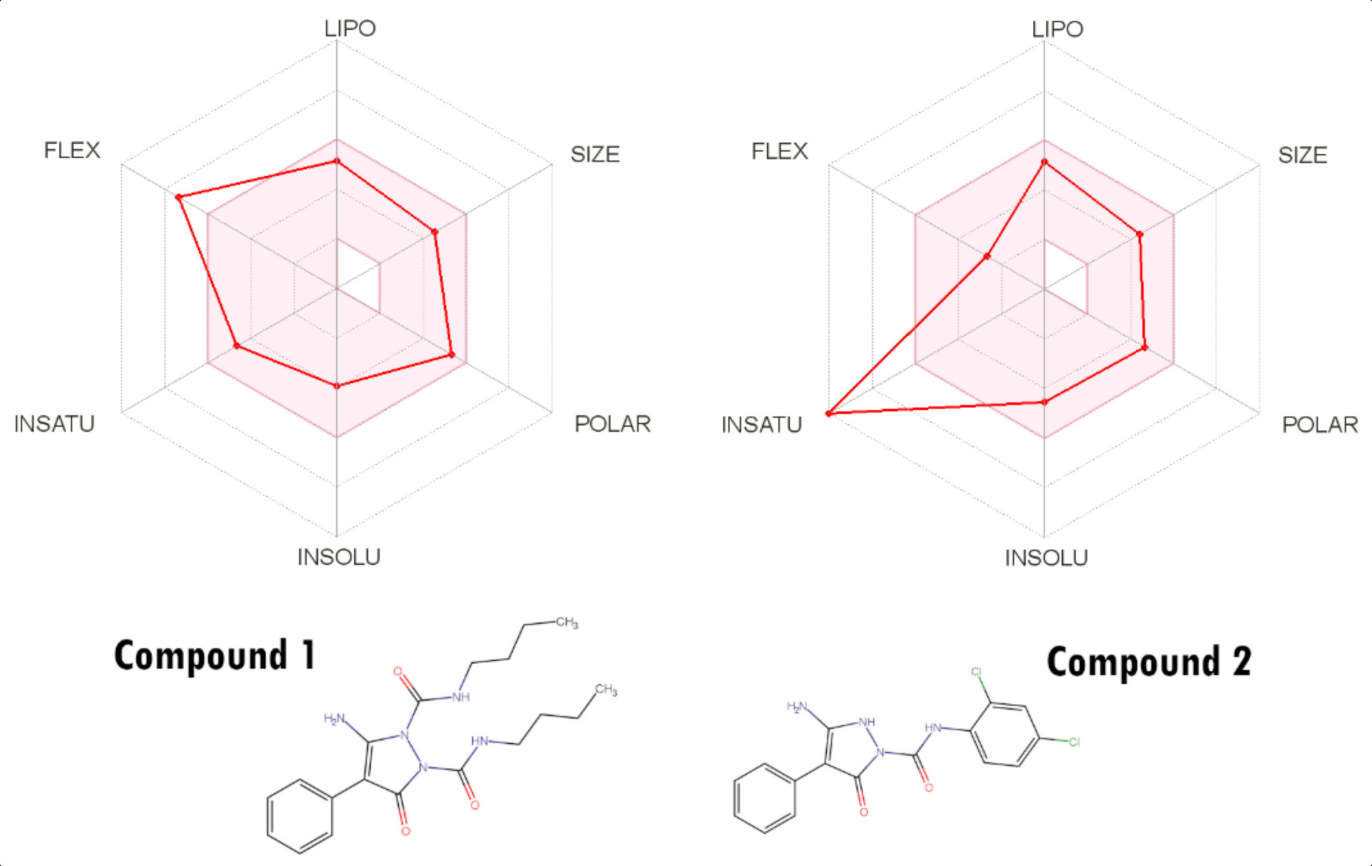
Oral bio-availability graph generated using the SwissADME service. The red--coloured zone is physicochemically suitable for oral bio-availability. LIPO—lipophility (−0.7 < XlogP3 < +5.0); SIZE—molecular weight (150 g/mol < MW < 500 g/mol); POLAR—polarity (20 Å^2^ < TPSA < 130 Å^2^); INSOLU—insolubility (0 < logS < 6); INSATU—insaturation (0.25 < fraction Csp3 < 1); FLEX—flexibility (0 < num. of rotatable bonds < 9).

The pharmacokinetic profile of compound **1** is very promising. ACD/Percepta software indicated optimal human plasma protein binding (74.49%); 4.6 L/kg of distribution volume, which means good distribution to all parts of the human body; and 91.7% of single 50 mg dose bio-availability per os. Compound **2** exhibited more problematic distribution properties: 96.81% human plasma protein binding, 0.34 L/kg of distribution volume, and 36.9% of single 50 mg dose bio-availability per os. Moreover, the prediction results (ACD/Percepta, admetSAR 2.0) indicate the possibility of blood–brain barrier penetration for both structures. The physicochemical profiles of compounds **1** and **2** indicate that both structures are good candidates for drug agents, as they both show the fulfillment of the Lipinski rule [19], the Ghose rule [20], the Egan rule [21], and the Muegge rule [22]. The basic physicochemical properties of compounds **1** and **2** were gathered in Table 5. The analysis results indicate that compounds can affect the pharmacokinetics of other drugs because of their effects on cytochromes P450 isoenzymes. Compound **1** showed inhibition properties for CYP2C19, CYP2C9, CYP3A4, and compound **2** showed inhibition properties for CYP1A2. Both compounds have very promising physicochemical properties for oral bio-availability (Figure 8). Both compounds showed a very low probability of positive AMES test results and hERG inhibition test results. A ProTox II analysis classified both compounds to toxicity class 4 (predicted LD_50_ 1000 mg/kg). Moreover, both compounds had very promising results of the detailed prediction of the toxicity profile—compound **1** showed a 0.6 probability of carcinogenicity (1 positive test result out of 17 different predictions), while compound **2** showed a 0.52 probability of carcinogenicity and a 0.64 probability of hepatotoxicity (2 positive test results out of 17 different predictions).

## 3. Materials and Methods

### 3.1. Chemistry

All of the chemicals used for the synthesis were purchased from Sigma-Aldrich, AlfaAesar, and POCH, and were used without further purification. The FTIR spectra were recorded with an IRTracer-100 Schimadzu Spectrometer (4000–600 cm^−1^), with an accuracy of recording 1 cm^−1^ using KBr pellets. The thermolysis of the compounds in the air atmosphere was studied using TG-DTG-DTA techniques in the temperature range of 25–1000 °C at a heating rate of 10 °C min^−1^; TG, DTG, and DTA curves were recorded on a Netzsch TG 209 apparatus under air atmosphere (v = 20 mL × min^−1^) using ceramic crucibles. Ceramic crucibles were also used as a reference material. All NMR experiments were run at 298 K on a 500 MHz Bruker Avance III spectrometer, which was equipped with ^1^H with a ^13^C BB probehead (^1^H-detected experiment) and operating at 500.13 and 125.76 MHz for ^1^H and ^13^C nuclei, respectively. The samples were prepared in DMSO-d_6_ (99.8% + D) from Armar Chemicals. The chemical shifts in ^1^H and ^13^C were referenced to the methyl groups of DMSO (2.50 and 39.5 ppm, respectively). The ^13^C NMR data were assigned by using the standard 2D ^1^H-^13^C NMR correlation techniques, gradient-selected heteronuclear single-quantum correlation (gs-HSQC) [23], and gradient-selected heteronuclear multiple-bond correlation (gs-HMBC) [24,25].

### 3.2. Crystal Structure Determination

X-ray data were collected at 100 K on an XtaLAB Synergy, Dualflex, Pilatus 300K diffractometer apparatus (Rigaku Corporation, Tokyo, Japan) equipped with a PhotonJet microfocus X-ray tube apparatus (Rigaku Corporation, Tokyo, Japan). Data reduction was performed using CrysAlisPro (Agilent Technologies UK Ltd., Yarnton, UK) [26]. The structure was refined in ShelXL [27]. Molecular plots and packing diagrams were drawn using Mercury [28]. Molecular geometry parameters were computed using PLATON and publCIF [29,30]. The crystallographic information files for the crystal structures are available under the deposition numbers: 2075915 and 2075918.

### 3.3. ADMET Analysis

Compound **1** and compound **2** were analyzed using ACDLabs Percepta software version 14.0.0 (Advanced Chemistry Development, Inc., Metropolitan, Toronto, ON, Canada), SwissADME service (Swiss Institute of Bioinformatics, Lausanne, Switzerland, 2021) [31], admetSAR 2.0 service (admetSAR 2019) [32], and ProTOX II service [33] to obtain the computational pharmacokinetic and toxicologic profiles of the tested compounds.

### 3.4. Biological Assays

To evaluate the active metabolic cells, the MTT (3-(4,5-dimethylthiazol-2-yl))-2,5 diphenyltetrazoliumbromide) assay was used [34]. In this method, EC_50_ (the effective concentration of the tested drug, where a 50% growth reduction is observed in cell growth compared to the untreated control) was used. An MTT assay was performed to test the in vitro cytotoxicity against the A549 cells, which were from a human lung adenocarcinoma that was obtained from the European Collection of Cell Cultures (ECACC, Salisbury, UK). The cells were cultured in Dulbecco’s Modified Eagle’s Medium (PAN-Biotech, Aidenbach, Germany), 100 units of penicillin/mL (Sigma Aldrich, St. Louis, MO, USA), 100 μg of streptomycin/mL (Sigma Aldrich, St. Louis, MO, USA), 2 mM L-glutamine (Sigma Aldrich, St. Louis, MO, USA), 10% Fetal Bovine Serum (FBS) (Sigma Aldrich, St. Louis, MO, USA), and MTT (3-(4,5-dimethylthiazol-2-yl))-2,5 diphenyltetrazoliumbromide) (Sigma Aldrich, St. Louis, MO, USA). To complete the analyses of the new compounds, the cells were cultured overnight at 37 °C with 5% CO_2_ in a standard 96-well flat-bottomed plate containing 10^4^ cells/well. The following day, the medium was replaced by 100 µL of **1**, **2,** and etoposide added in varying concentrations to the wells. After 24 h of incubation, 50 µL MTT was added to each well for the last 2 h. The final absorbance was measured in analytical wavelengths (570 nm for blue-violet insoluble formazan) using a microplate reader (Synergy H1, BioTek, Winooski, VT, USA). The viability and cell cycle analysis results were presented as mean ± standard deviation. All assays were performed in triplicate, and the results were obtained in three independent experiments [35].

## 4. Conclusions

In summary, etoposide is one of the most commonly used anticancer agents. For many years, it has been the standard therapy for small cell lung cancer, leukemia, lymphoma, germ-cell tumors, and neuroblastoma [36]. Our present findings have shown that both derivatives were very effective in the inhibition of human lung adenocarcinoma cell growth in comparison with etoposide. In conclusion, our present findings showed that the new 3-amino-N-(2,4-dichlorophenyl)-5-oxo-4-phenyl-2,5-dihydro-1H-pyrazole-1-carboxamide **2** (EC_50_ = 220.20+/−22.47 µM,) was much more effective in the inhibition of human lung adenocarcinoma cell growth in comparison to compound **1** with 2,4-dichlorophenyl moiety. These results suggest that compound **2** may be a promising molecule for the treatment of lung cancer. In addition, our studies gained new knowledge about pyrazole derivatives.

## Figures and Tables

**Table 1:** Crystal and structure refinement data for **1** and **2** compounds.

Compound	1	2
Chemical formula	C_19_H_27_N_5_O_3_	C_16_H_12_Cl_2_N_4_O_2_·2(C_2_H_6_OS)·H_2_O
*M*_r_	373.45	537.47
Crystal system, space group	Monoclinic, *P*2_1_/*c*	Monoclinic, *P*2_1_/*c*
Temperature (K)	100	100
*a*, *b*, *c* (Å)	11.9591 (2), 16.2222 (2), 11.6359 (2)	7.0863 (2), 25.7334 (8), 13.7460 (4)
β (°)	117.695 (2)	97.800 (3)
*V* (Å^3^)	1998.78 (6)	2483.45 (13)
*Z*	4	4
Radiation type	Cu *K*α	Mo *K*α
No. of measured, independent and observed [*I* > 2σ(*I*)] reflections	24,501, 4143, 3729	37,018, 7334, 5953
*R*_int_	0.039	0.078
(sin θ/λ)_max_ (Å^−1^)	0.635	0.736
*R*[*F*^2^ > 2σ (*F*^2^)], *wR*(*F*^2^), *S*	0.044, 0.119, 1.03	0.079, 0.203, 1.15
No. of reflections	4143	7334
No. of parameters	266	337

**Table 2:** Hydrogen-bond geometry (Å, °) for **1**.

*D*—H⋯*A*	*D*—H	H⋯*A*	*D*⋯*A*	*D*—H⋯*A*
N26—H26*A*⋯O25 ^i^	0.86	1.99	2.8055 (16)	158
N26—H26*B*⋯O18	0.86	2.11	2.7088 (17)	127
N20—H20⋯O27	0.86	2.00	2.6844 (15)	136
N13—H13⋯O27 ^ii^	0.86	2.00	2.8210 (15)	160

Symmetry codes: (i) *x*, −*y* + 3/2, *z* − 1/2; (ii) −*x* + 1, −*y* + 1, −*z* + 1.

**Table 3:** Hydrogen-bond geometry (Å, °) for **2**.

*D*—H⋯*A*	*D*—H	H⋯*A*	*D*⋯*A*	*D*—H⋯*A*
N1—H1⋯O10	0.86	1.97	2.689 (3)	140 S(6)
N5—H5⋯S1*R*	0.86	2.66	3.459 (3)	155
N5—H5⋯O1*R*	0.86	2.26	2.799 (4)	121
N7—H7*A*⋯O1*W* ^i^	0.86	2.01	2.835 (4)	160
N7—H7*B*⋯O1Aa	0.86	2.06	2.81 (4)	146
N7—H7*B*⋯O1Bb	0.86	2.17	2.86 (3)	137
O1*W*—H1*W*1⋯O1*R*	0.81 (2)	2.02 (3)	2.817 (4)	166 (7)
O1*W*—H1*W*2⋯O3 ^ii^	0.81 (2)	2.08 (3)	2.867 (3)	163 (6)

Symmetry codes: (i) −x + 2, −y + 1, −z + 2 (ii) x + 1, y, z.

**Table 4:** Cytotoxicity activity at the EC_50_.

Compound	Cytotoxicity Activity EC_50_ [μM]
**1**	613.22 +/−23.56
**2**	220.20+/−22.47
etoposide	451.47+/−18.27 *

Results are presented as the means ± SD; EC_50_, 50% inhibition of the cell viability. Statistical significance was assessed using a one-way ANOVA analysis. * *p* < 0.01 was considered significantly different between cancer and non-cancer cell lines.

**Table 5:** Basic physicochemical properties of the two compounds.

Compound	Molecular Weight [g/mol]	LogP	pKa (Acid)	pKa (Base)	TPSA [Å^2^]	Molar Refractivity [m^3^/mol]
**1**	373.45	2.83	11.70	3.90	111.15	106.29
**2**	363.20	3.17	9.60	3.48	92.91	95.59

Log *p* value is an average of 5 prediction algorithms (iLOGP, XLOGP, WLOGP, MLOGP, SILICOS-IT); TPSA—topological polar surface area.

## Data Availability

The conducted research was presented for the first time in this publication.

## Supplementary Materials

The following are available online at https://www.mdpi.com/article/10.3390/ijms22136692/s1. Figure S1. Solution-state 1H NMR (DMSO-d6) of **1** (a) and **2** (b). Figure S2. Solution-state 13C NMR (DMSO-d6) of **1** (a) and **2** (b).

## References

[1] Pitucha M., Rzymowska J. (2012). Anticancer screening and structure activity relationship study of some semicarbazides and 1,2,4-Triazolin-5-ones. Lett. Drug Des. Discov..

[2] Kumar V., Kaur K., Gupta G.K., Sharma A.K. (2013). Pyrazole containing natural products: Synthetic preview and biological significance. Eur. J. Med. Chem..

[3] Thawabteh A., Juma S., Bader M., Karaman D., Scrano L., Bufo S.A., Karaman R. (2019). The biological activity of natural alkaloids against herbivores, cancerous cells and pathogens. Toxins.

[4] Dai H., Xiao Y.S., Li Z., Xu X.Y., Qian X.H. (2014). The thiazoylmethoxy modification on pyrazole oximes: Synthesis and insecticidal biological evaluation beyond acaricidal activity. Chin. Chem. Lett..

[5] Saundane A.R., Verma V.A., Katkar V.T. (2013). Synthesis and antimicrobial and antioxidant activities of some new 5-(2-Methyl-1 H -indol-3-yl)-1,3,4-oxadiazol-2-amine derivatives. J. Chem..

[6] El-Borai M.A., Rizk H.F., Sadek M.R., El-Keiy M.M. (2016). An eco-friendly synthesis of heterocyclic moieties condensed with pyrazole system under green conditions and their biological activity. Green Sustain. Chem..

[7] Yang B., Lu Y., Chen C.J., Cui J.P., Cai M.S. (2009). The synthesis of 5-amino-4-arylazo-3-methyl-1H-pyrazoles and 5-aryl-3-methylpyrazole[3,4-e] [1–4] tetrazines. Dyes Pigment..

[8] Sener N., Sener I., Yavuz S., Karci F. (2015). Synthesis, absorption properties and biological evaluation of some novel disazo dyes derived from pyrazole derivatives. Asian J. Chem..

[9] Abdel-Mohsen S.A., El-Emary T.I. (2018). New pyrazolo [3,4-b] pyridines: Synthesis and antimicrobial Activity. Der Pharma Chem..

[10] El-Dean A.M.K., Zaki R.M., Geies A.A., Radwan S.M., Tolba M.S. (2013). Synthesis and antimicrobial activity of new heterocyclic compounds containing thieno[3,2-c]coumarin and pyrazolo[4,3-c]coumarin frameworks. Russ. J. Bioorgan. Chem..

[11] Skehan P., Storeng R., Scudiero D., Monks A., Mcmahon J., Vistica D., Warren J.T., Bokesch H., Kenney S., Boyd M.R. (1990). New colorimetric cytotoxicity assay for anticancer-drug screening. J. Natl. Cancer Inst..

[12] Brand-Williams W., Cuvelier M.E., Berset C. (1995). Use of a free radical method to evaluate antioxidant activity. LWT Food Sci. Technol..

[13] Liu X.H., Qiao L., Zhai Z.W., Cai P.P., Cantrell C.L., Tan C.X., Weng J.Q., Han L., Wu H.K. (2019). Novel 4-pyrazole carboxamide derivatives containing flexible chain motif: Design, synthesis and antifungal activity. Pest. Manag. Sci..

[14] Yan Z., Liu A., Huang M., Liu M., Pei H., Huang L., Yi H., Liu W., Hu A. (2018). Design, synthesis, DFT study and antifungal activity of the derivatives of pyrazolecarboxamide containing thiazole or oxazole ring. Eur. J. Med. Chem..

[15] Ren Z.L., Liu H., Jiao D., Hu H.T., Wang W., Gong J.X., Wang A.L., Cao H.Q., Lv X.H. (2018). Design, synthesis, and antifungal activity of novel cinnamon–pyrazole carboxamide derivatives. Drug Dev. Res..

[16] Isyaku Y., Uzairu A., Uba S. (2020). In silico studies of novel pyrazole-furan and pyrazole-pyrrole carboxamide as fungicides against Sclerotinia sclerotiorum. Beni Suef Univ. J. Basic Appl. Sci..

[17] Kaczor A.A., Wrobel T., Karczmarzyk Z., Wysocki W., Fruzinski A., Brodacka M., Matosiuk D., Pitucha M. (2014). Structural studies on N-(1-naphthyl)-3-amino-5-oxo-4-phenyl-1hpyrazole-1-carboxamide with antibacterial activity. Lett. Organ. Chem..

[18] Bernstein J., Davis R.E., Shimoni L., Chang N.-L. (1995). Patterns in hydrogen bonding: Functionality and graph set analysis in crystals. Angew. Chem. Int. Ed. Engl..

[19] Lipinski C.A., Lombardo F., Dominy B.W., Feeney P.J. (2001). Experimental and computational approaches to estimate solubility and permeability in drug discovery and development settings. Adv. Drug Deliv. Rev..

[20] Ghose A.K., Viswanadhan V.N., Wendoloski J.J. (1999). A knowledge-based approach in designing combinatorial or medicinal chemistry libraries for drug discovery. 1. A qualitative and quantitative characterization of known drug databases. J. Comb. Chem..

[21] Egan W.J., Merz K.M., Baldwin J.J. (2000). Prediction of drug absorption using multivariate statistics. J. Med. Chem..

[22] Muegge I., Heald S.L., Brittelli D. (2001). Simple selection criteria for drug-like chemical matter. J. Med. Chem..

[23] Bodenhausen G., Ruben D.J. (1980). Natural abundance nitrogen-15 NMR by enhanced heteronuclear spectroscopy. Chem. Phys. Lett..

[24] Bax A., Summers M.F. (1986). Proton and carbon-13 assignments from sensitivity-enhanced detection of heteronuclear multiple-bond connectivity by 2D multiple quantum NMR. J. Am. Chem. Soc..

[25] Willker W., Leibfritz D., Kerssebaum R., Bermel W. (1993). Gradient selection in inverse heteronuclear correlation spectroscopy. W. Magn. Reson. Chem..

[26] Agilent Technologies UK (2017). CrysAlisPro 1.171.39.33c.

[27] Sheldrick G.M. (2015). SHELXT—Integrated space-group and crystal-structure determination. Acta Crystallogr. Sect. C Struct. Chem..

[28] Macrae C.F., Bruno I.J., Chisholm J.A., Edgington P.R., Mccabe P., Pidcock E., Rodriguez–Monge L., Taylor R., Van De Streek J., Wood P.A. (2008). Mercury: Visualization and analysis of crystal structures. J. Appl. Cryst..

[29] Spek A.L. (2009). Structure validation in chemical crystallography. Acta Crystallogr. Sect. D Biol. Crystallogr..

[30] Westrip S.P. (2010). publCIF: Software for editing, validating and formatting crystallographic information files. J. Appl. Cryst..

[31] Daina A., Michielin O., Zoete V. (2017). SwissADME: A free web tool to evaluate pharmacokinetics, drug-likeness and medicinal chemistry friendliness of small molecules. Sci. Rep..

[32] Yang H.B., Lou C.F., Sun L.X., Li J., Cai Y.C., Wang Z., Li W.H., Liu G.X., Tang Y. (2019). admetSAR 2.0: Web-service for prediction and optimization of chemical ADMET properties. Bioinformatics.

[33] Banerjee P., Eckert A.O., Schrey A.K., Preissner R. (2018). ProTox-II: A webserver for the prediction of toxicity of chemicals. Nucleic Acids Res..

[34] Plumb J.A., Langdon S.P. (2004). Cell sensitivity assays: The MTT assay. Cancer Cell Culture: Methods and Protocols.

[35] Girek M., Kłosiński K., Grobelski B., Pizzimenti S., Cucci M.A., Daga M., Barrera G., Pasieka Z., Czarnecka K., Szymański P. (2019). Novel tetrahydroacridine derivatives with iodobenzoic moieties induce G0/G1 cell cycle arrest and apoptosis in A549 non-small lung cancer and HT-29 colorectal cancer cells. Mol. Cell. Biochem..

[36] Mascaux C., Paesmans M., Berghmans T., Branle F., Lafitte J.J., Lemaitre F., Meert A.P., Vermylen P., Sculier J.P. (2000). European Lung Cancer Working Party (ELCWP). A systematic review of the role of etoposide and cisplatin in the chemotherapy of small cell lung cancer with methodology assessment and meta-analysis. Lung Cancer.

