# Supplementary material for: Cytotoxic Activity against A549 Human Lung Cancer Cells and ADMET Analysis of New Pyrazole Derivatives
Source: Int J Mol Sci. 2021 Jun 22;22(13):6692. doi: 10.3390/ijms22136692 (PMC8269030; doi:10.3390/ijms22136692)
Supplement: Supplementary file 1 [file ijms-22-06692-s001.zip › ijms-1257581-supplementary.pdf]

International Journal of
Molecular Sciences

MDPI

Article

Cytotoxic activity against A549 human lung cancer cells and ADMET analysis of new pyrazole derivatives

Agnieszka Czylkowska [1,*], Małgorzata Szczesio [1], Anita Raducka [1], Bartłomiej Rogalewicz [1], Paweł Kręcisz [2], Kamila Czarnecka [2,3], Paweł Szymański [2,3], Monika Pitucha [4], and Tomasz Pawlak [5]

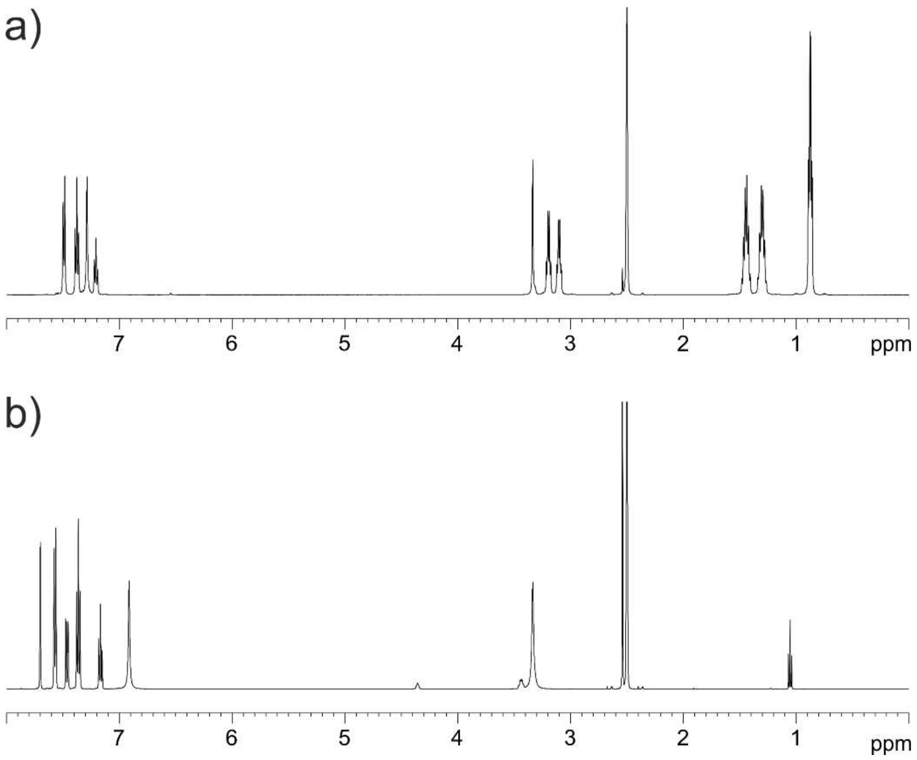

Figure S1. Solution-state ^{1}H NMR (DMSO-d6) of **1** (a) and **2** (b).

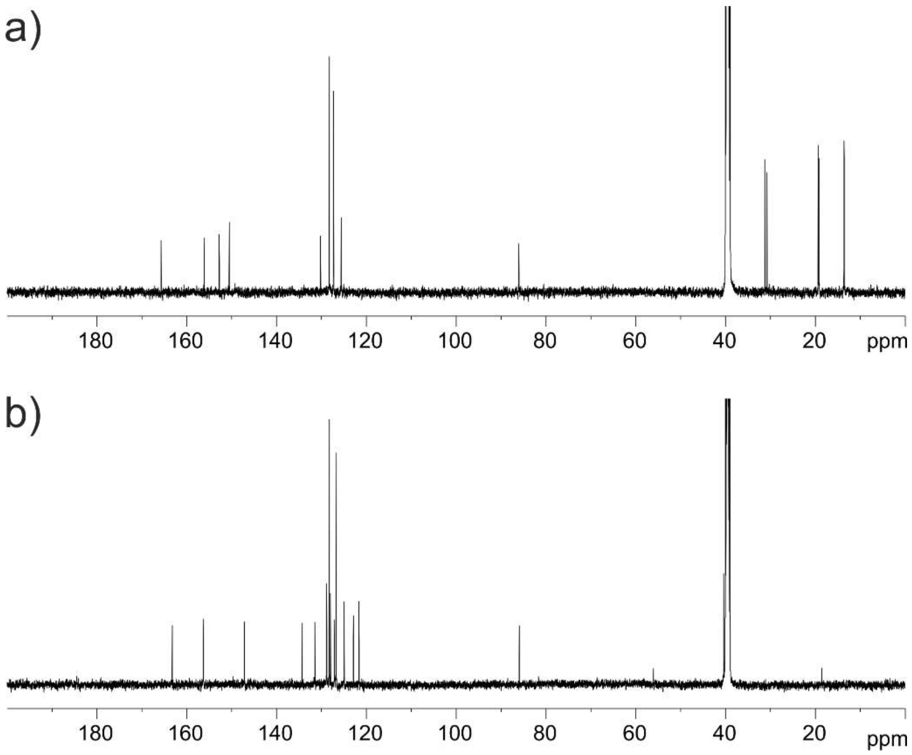

Figure S2. Solution-state ^{13}C NMR (DMSO-d6) of **1** (a) and **2** (b).
